# Supplementary material for: Dietary glucosamine overcomes the defects in αβ-T cell ontogeny caused by the loss of de novo hexosamine biosynthesis
Source: Nat Commun. 2022 Dec 1;13:7404. doi: 10.1038/s41467-022-35014-w (PMC9715696; doi:10.1038/s41467-022-35014-w)
Supplement: Supplementary file 1 — Supplementary Information [file 41467_2022_35014_MOESM1_ESM.pdf]

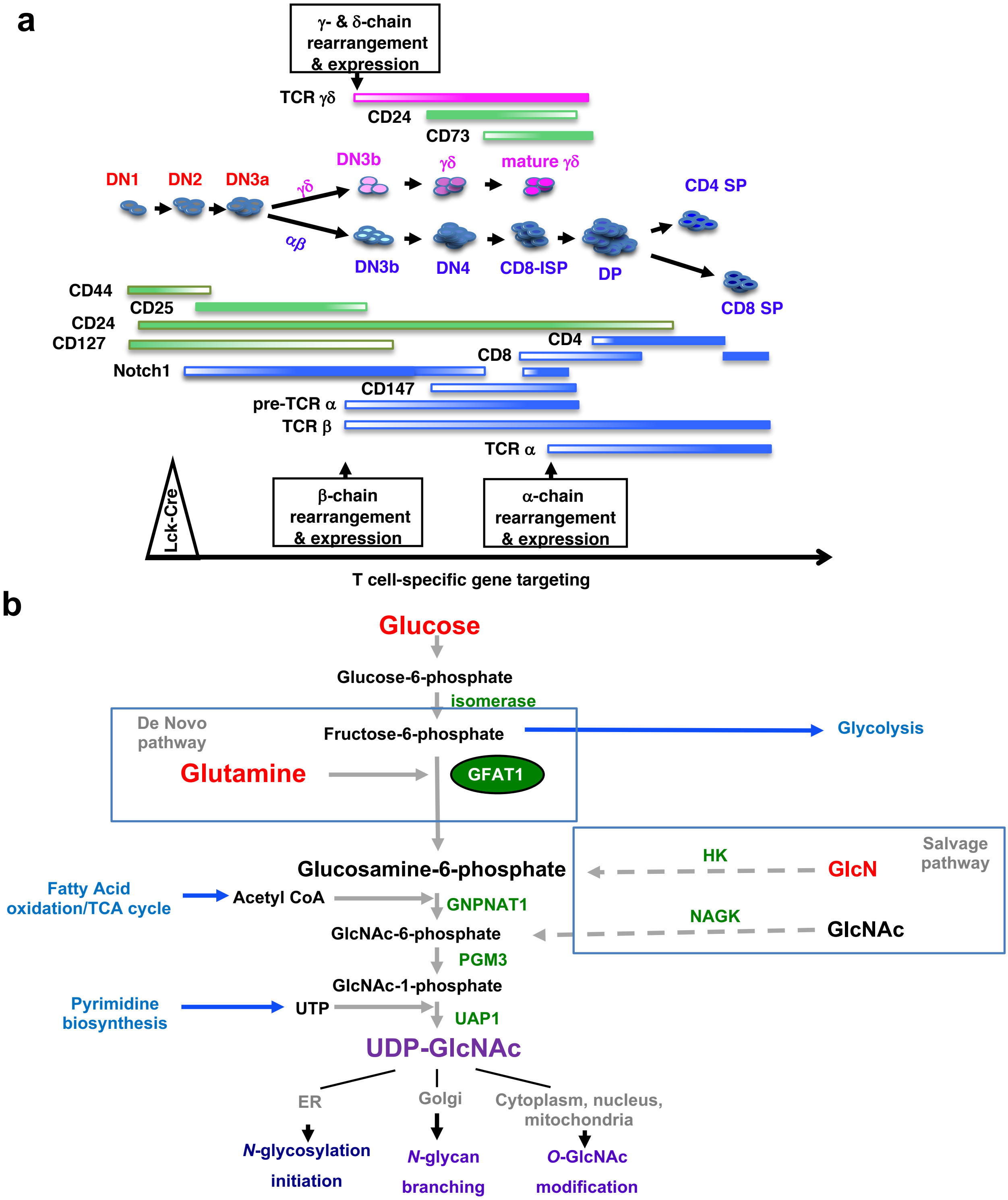

Supplementary Figure 1. Cartoon of T cell development in the thymus with concurrent T cell-specific gene deletion and cartoon of the Hexosamine Biosynthesis Pathway (HBP).

a. Two distinct T cell lineages arise from a double negative (DN; $CD4^-CD8^-Lin^-$) precursor. Rearrangements of the TCR subunit genes begin at the DN2 stage and divergence of lineages (αβ vs γδ) are thought to be complete by the DN3 stage. Expression of cell surface receptors/markers at the different stages are indicated with a bar. Blue bars represent markers effective only in cells of the αβ-lineage, pink bars present only in cells of the γδ-lineage and green bars are common to both. Gradient of color in bars represent increasing/decreasing expression. (DN double negative; ISP immature single positive; DP double positive; SP single positive). Owing to the stage-specific expression of the tyrosine kinase, Lck, T cell-specific gene deletion starts at the early DN2 stage and lasts throughout the entire phase of thymocyte development and mature T cells **b.** Schematic diagram of the hexosamine biosynthesis pathway. The enzymes along the pathway are indicated in green. GFAT1: glutamine:fructose-6-phosphate amidotransferase 1, GNPNAT1: glucosamine-phosphate *N*-acetyltransferase 1, PGM3: phosphoglucomutase 3, UAP1: UDP *N*-acetylhexosamine pyrophosphorylase 1. HB Salvage pathway: HK: hexokinase, NAGK: *N*-acetyl-*D*-glucosamine kinase. GlcN: glucosamine, GlcNAc: *N*-acetylglucosamine.

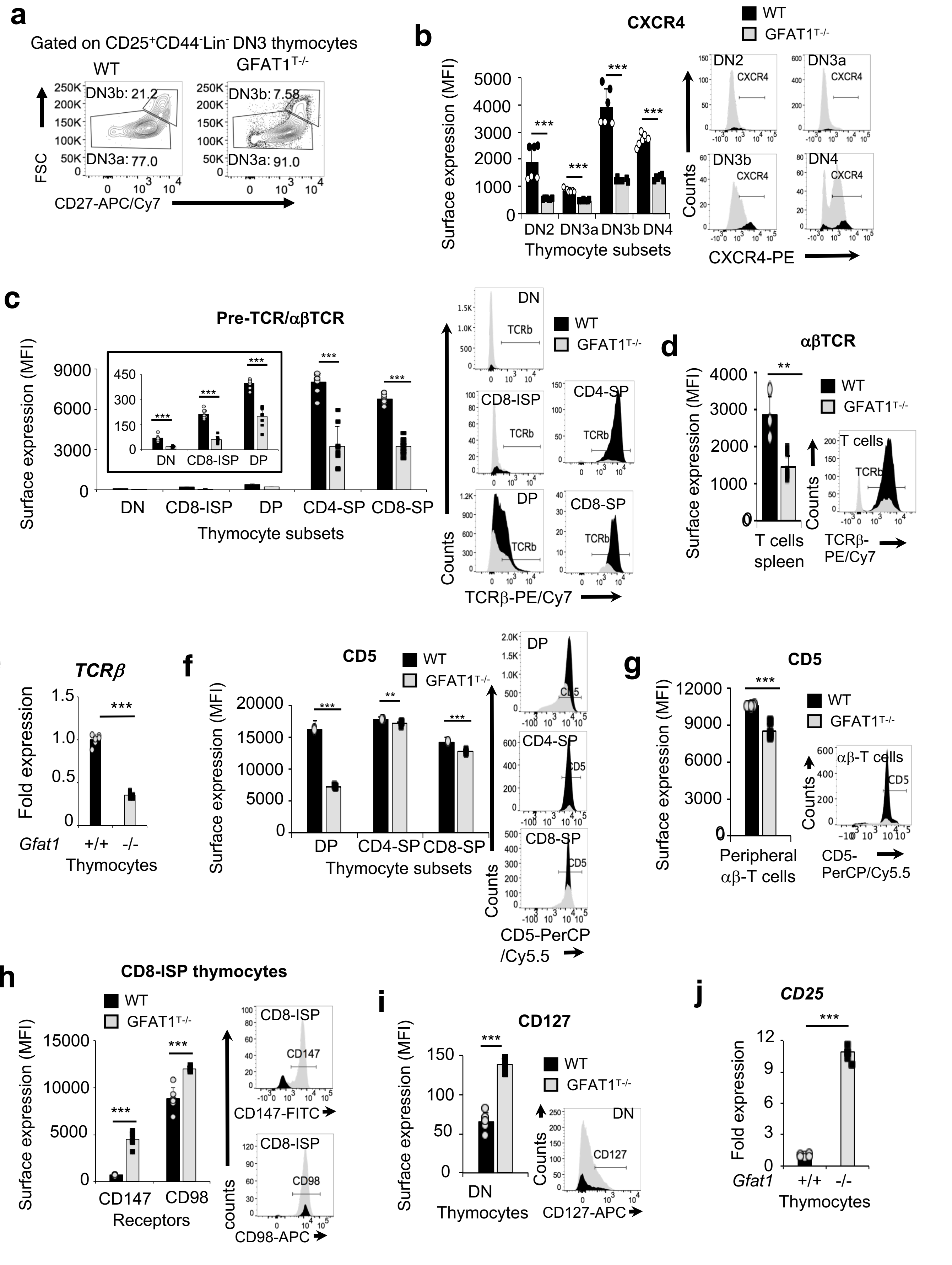
a
Gated on CD25+CD44-Lin- DN3 thymocytes
WT
GFAT1T-/-
DN3b: 21.2
DN3a: 77.0
DN3b: 7.58
DN3a: 91.0
FSC
CD27-APC/Cy7
b
CXCR4
WT
GFAT1T-/-
Surface expression (MFI)
DN2 DN3a DN3b DN4
Thymocyte subsets
DN2
DN3a
DN3b
DN4
Counts
CXCR4-PE
c
Pre-TCR/αβTCR
Surface expression (MFI)
DN CD8-ISP DP CD4-SP CD8-SP
Thymocyte subsets
TCRb
Counts
TCRβ-PE/Cy7
d
αβTCR
T cells spleen
T cells
TCRβ-PE/Cy7
e
TCRβ
Fold expression
Gfat1 +/+ -/-
Thymocytes
f
CD5
DP CD4-SP CD8-SP
Thymocyte subsets
CD5-PerCP/Cy5.5
g
CD5
Peripheral αβ-T cells
αβ-T cells
CD5-PerCP/Cy5.5
h
CD8-ISP thymocytes
CD147 CD98
Receptors
CD147-FITC
CD98-APC
i
CD127
DN
Thymocytes
CD127-APC
j
CD25
Fold expression
Gfat1 +/+ -/-
Thymocytes

Supplementary Figure 2. Decreased proportion of CD27-expressing DN3b thymocytes and defective expression of cell surface receptors occur in the absence of GFAT1.

Thymocytes or splenocytes from male and female wild type (WT) and GFAT1$^{T-/-}$ littermates were harvested and stained for CD4, CD8α, CD3ε, NK1.1, B220, Ter119, Gr1, CD25, CD27, CD44, CXCR4, CD127 and analyzed by flow cytometry. **a** The relative amounts of Lin^{-}CD25^{+}CD44^{-}CD27^{low} DN3a and Lin^{-}CD25^{+}CD44^{-}CD27^{+}FCS^{+} DN3b subsets are indicated. FSC = forward scatter. FACS plots are representative of three experiments with similar results. **b** Bar graph represents median fluorescence intensity (MFI) of CXCR4 on the surface of the indicated DN subsets (n=6 mice each). Representative FACS plots of CXCR4 staining are shown. **c-d, f-h**. Thymocytes or splenocytes were stained with CD4, CD8α, CD147, CD5, CD98, TCRβ and analyzed by flow cytometer. c-d Bar graphs represent median fluorescence intensity (MFI) of surface TCRβ expression on thymocytes (n=8-9 mice each)(c) (Inset is a blowup of the expression on DN, CD8-ISP and DP stages), or surface TCRβ expression on peripheral T cells (n= 3 mice each)(d). FACS plots of TCRβ staining are shown on the right side of the bar graphs. f-g Bar graphs represent median fluorescence intensity (MFI) of CD5 on the surface of DP and SP thymocytes (n=3 mice each)(f) or peripheral T cells (n=6 mice each)(g). Representative FACS plots of CD5 staining are shown. h Bar graphs represent median fluorescence intensity (MFI) of CD147 and CD98 on the surface of CD8-ISP cells (n=6-8 mice each). Representative FACS plots of CD147 and CD98 staining are shown. i Bar graph represents median fluorescence intensity (MFI) of CD127 on the surface of DN cells (n=6 mice each). Representative FACS plot of CD127 staining is shown. **e, j.** Thymocytes were harvested from 5-wk old male and female WT or GFAT1$^{T-/-}$ littermates. RNA was extracted and subjected to qRT-PCR analysis. Bar graph indicates fold expression relative to wt. n=6 independent samples. All data (a-j) from bar graphs denote mean $\pm$ SD. $^{**}p < 0.01$, $^{***}p < 0.001$ using two-sided Student's *t*-test. Representative FACS plots (a-d, f-i) are from at least three experiments with similar results. Source data are available for a-j.

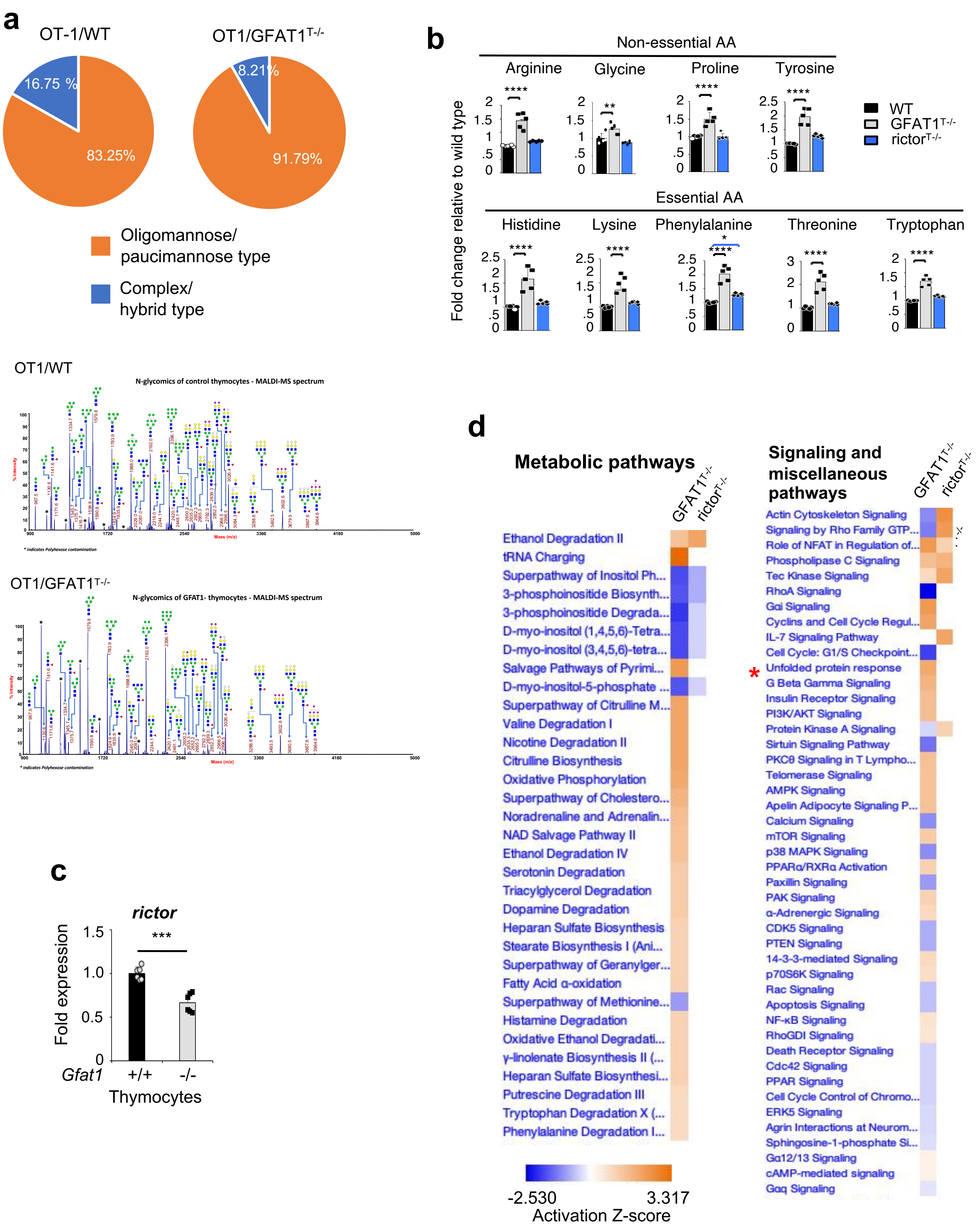
a
OT-1/WT
OT1/GFAT1T-/-
16.75 %
83.25%
8.21%
91.79%
Oligomannose/ paucimannose type
Complex/ hybrid type
OT1/WT
N-glycomics of control thymocytes - MALDI-MS spectrum
% Intensity
Mass (m/z)
* indicates Polyhexose contamination
OT1/GFAT1T-/-
N-glycomics of GFAT1- thymocytes - MALDI-MS spectrum
% Intensity
Mass (m/z)
* indicates Polyhexose contamination
b
Non-essential AA
Arginine
Glycine
Proline
Tyrosine
Essential AA
Histidine
Lysine
Phenylalanine
Threonine
Tryptophan
Fold change relative to wild type
WT
GFAT1T-/-
rictorT-/-
c
rictor
Fold expression
Gfat1
+/+
-/-
Thymocytes
d
Metabolic pathways
GFAT1T-/-
rictorT-/-
Ethanol Degradation II
tRNA Charging
Superpathway of Inositol Ph...
3-phosphoinositide Biosynth...
3-phosphoinositide Degrada...
D-myo-inositol (1,4,5,6)-Tetra...
D-myo-inositol (3,4,5,6)-tetra...
Salvage Pathways of Pyrimi...
D-myo-inositol-5-phosphate ...
Superpathway of Citrulline M...
Valine Degradation I
Nicotine Degradation II
Citrulline Biosynthesis
Oxidative Phosphorylation
Superpathway of Cholestero...
Noradrenaline and Adrenalin...
NAD Salvage Pathway II
Ethanol Degradation IV
Serotonin Degradation
Triacylglycerol Degradation
Dopamine Degradation
Heparan Sulfate Biosynthesis
Stearate Biosynthesis I (Ani...
Superpathway of Geranylger...
Fatty Acid α-oxidation
Superpathway of Methionine...
Histamine Degradation
Oxidative Ethanol Degradati...
γ-linolenate Biosynthesis II (...
Heparan Sulfate Biosynthesi...
Putrescine Degradation III
Tryptophan Degradation X (...
Phenylalanine Degradation I...
-2.530
3.317
Activation Z-score
Signaling and miscellaneous pathways
GFAT1T-/-
rictorT-/-
Actin Cytoskeleton Signaling
Signaling by Rho Family GTP...
Role of NFAT in Regulation of...
Phospholipase C Signaling
Tec Kinase Signaling
RhoA Signaling
Gαi Signaling
Cyclins and Cell Cycle Regul...
IL-7 Signaling Pathway
Cell Cycle: G1/S Checkpoint...
Unfolded protein response
G Beta Gamma Signaling
Insulin Receptor Signaling
PI3K/AKT Signaling
Protein Kinase A Signaling
Sirtuin Signaling Pathway
PKCθ Signaling in T Lympho...
Telomerase Signaling
AMPK Signaling
Apelin Adipocyte Signaling P...
Calcium Signaling
mTOR Signaling
p38 MAPK Signaling
PPARα/RXRα Activation
Paxillin Signaling
PAK Signaling
α-Adrenergic Signaling
CDK5 Signaling
PTEN Signaling
14-3-3-mediated Signaling
p70S6K Signaling
Rac Signaling
Apoptosis Signaling
NF-κB Signaling
RhoGDI Signaling
Death Receptor Signaling
Cdc42 Signaling
PPAR Signaling
Cell Cycle Control of Chromo...
ERK5 Signaling
Agrin Interactions at Neurom...
Sphingosine-1-phosphate Si...
Gα12/13 Signaling
cAMP-mediated signaling
Gαq Signaling

Supplementary Figure 3 Defects in *N*-glycans, metabolic and signaling pathways occur during GFAT1 deficiency.

a Thymocytes from 5-6 wk old male and female OT-1 or OT-1/GFAT1$^{T-/-}$ mice were harvested and processed for *N*-glycan analysis by mass spectrometry. The *N*-glycan structures were assigned using Glycoworkbench software based on precursor masses (Sodiated) and common mammalian biosynthetic pathways. Pie chart shows relative abundance of oligomannose/paucimannose-type versus complex/hybrid type glycans (upper panel). MALDI/MS spectra indicating *N*-glycan profile of OT1/WT vs OT1/GFAT1$^{T-/-}$ are shown below. **b** Thymocytes from age-matched male and female WT, GFAT1$^{T-/-}$ or rictor$^{T-/-}$ mice were harvested and counted. Metabolites were extracted and analyzed from equivalent thymocyte numbers by LC/MS. Bar graphs represent mean fold changes of indicated metabolite relative to WT (n=5 independent samples each). Error bars denote SD. *p < 0.05, **p < 0.01, ***p < 0.001, ****p < 0.0001 using one-way ANOVA followed by Tukey's *post-hoc* test. **c** Thymocytes were harvested from 5-wk-old male and female WT or GFAT1$^{T-/-}$ littermates. RNA was extracted and subjected to qRT-PCR. n=6 independent samples. Data are mean $\pm$ SD. ***p < 0.001 using two-sided Student's *t*-test. **d** Thymocytes from male and female WT, GFAT1$^{T-/-}$ or rictor$^{T-/-}$ mice were harvested and lysed in RIPA buffer. Cell lysates were subjected to quantitative proteomics by LC-MS/MS and data were analyzed by IPA. Heat map of statistically significant (p<0.05 as determined using two-sided Student's *t*-test) canonical metabolic and signaling pathway alterations in GFAT1- and rictor-deficient thymocytes relative to WT is shown. Red asterisk denotes increased UPR. Pathways are ranked according to the z-score that predicts upregulation (orange) or downregulation (blue).

Source data are available for b-c.

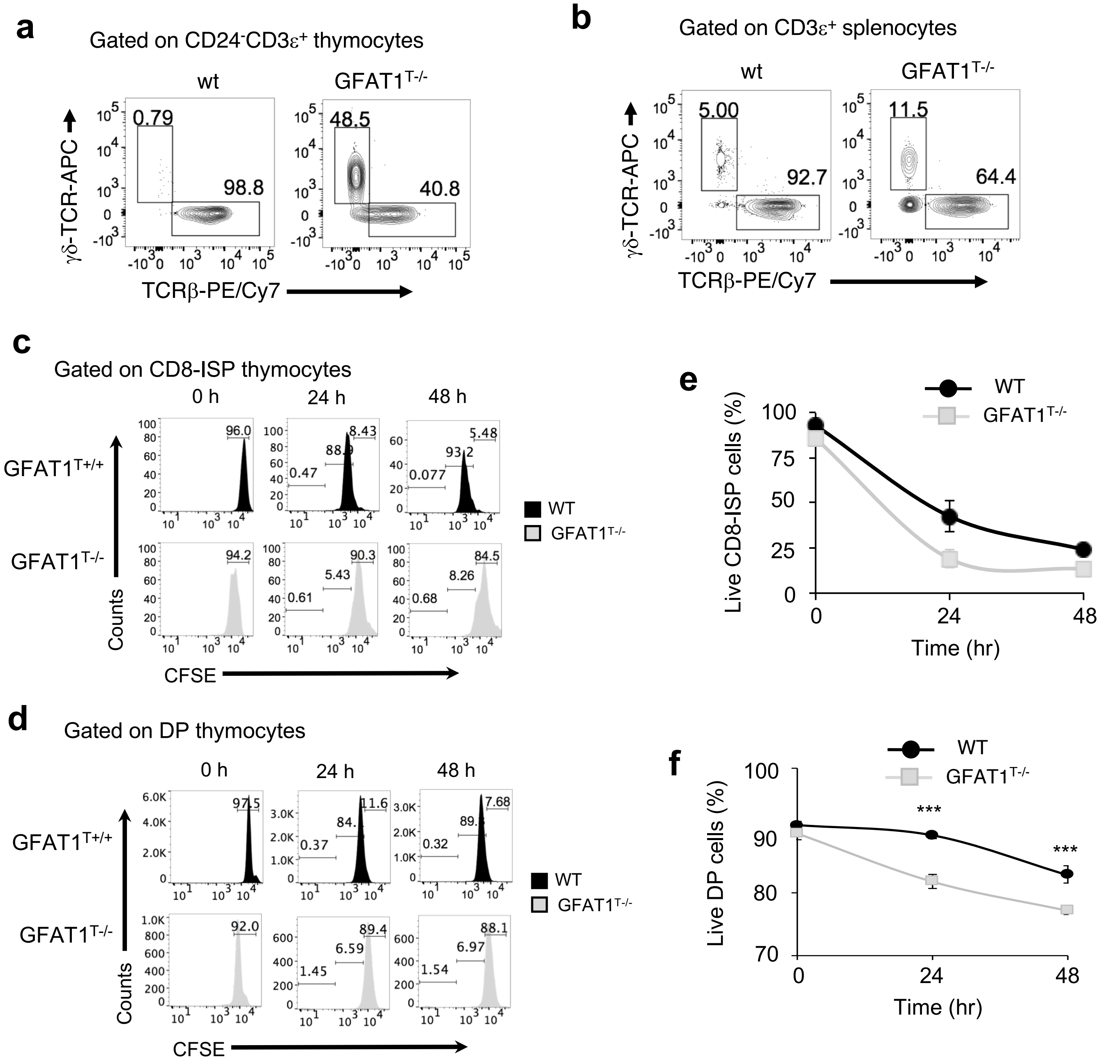

Supplementary Figure 4 GFAT1 deficiency favor γδ-T cell development.
a Thymocytes from age-matched WT and GFAT1$^{T-/-}$ mice were harvested and stained for CD4, CD8α, CD3ε, CD24, γδTCR and TCRβ and analyzed by flow cytometer. FACS plots show relative amounts of CD24^{-}CD3ε^{+} subsets expressing either γδTCR or TCRβ. Shown is a representative experiment out of 3 with similar results. **b.** Splenocytes were harvested from age-matched WT and GFAT1$^{T-/-}$ mice, stained for CD3ε, CD4, CD8α, TCRβ and γδTCR expression followed by flow cytometric analysis. FACS plots show proportion of αβ- and γδ T cells among CD3ε^{+} splenocytes are shown as revealed by surface expression of TCRβ or γδTCR. Shown is a representative experiment out of 3 with similar results. **c-d.** Thymocytes were labeled with CFSE and cultured *ex vivo* for 24h or 48 hr in complete DMEM media. Cells were harvested and stained for CD4, CD8α, CD3ε, CD147 and TCRβ prior to measuring the fluorescence of CFSE in CD8-ISP (c) or DP (d) cells. The fluorescence intensity decreases with each round of cell division and the corresponding percentage of cells is indicated in each plot. Shown is one representative experiment out of 3 with similar results. **e-f.** Age-matched WT and GFAT1$^{-/-}$ thymocytes were cultured *ex vivo* for 24h or 48 hr in complete DMEM media, harvested and stained for CD4, CD8α, CD147, Annexin V and TCRβ. Viability of CD8-ISP (n=4 mice each)(e) and DP (n=4 mice each)(f) cells was measured by flow cytometry and plotted. Data are mean ± SD. *$p < 0.05$, **$p < 0.01$, ***$p < 0.001$ using two-sided Student's *t*-test.

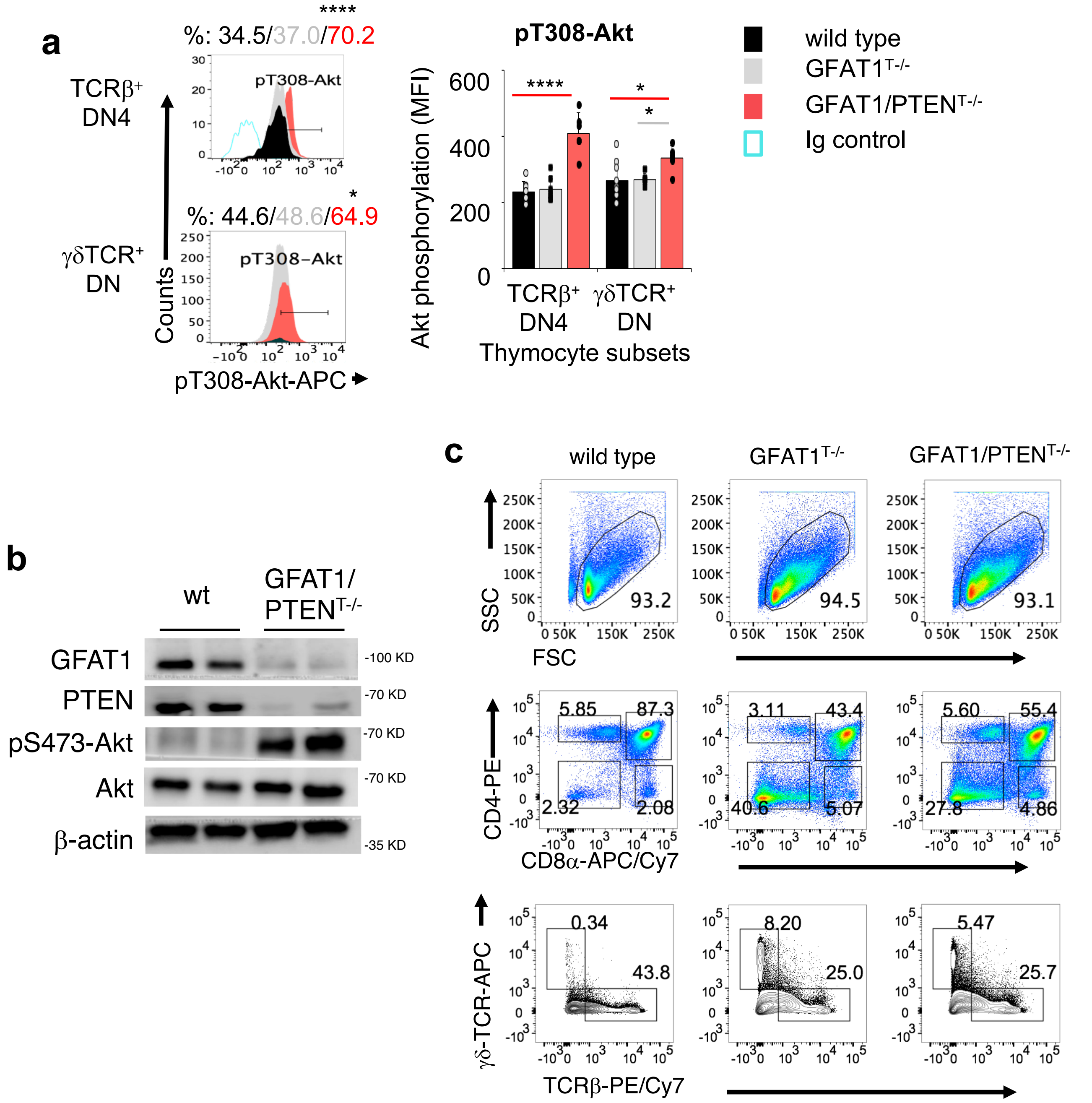

Supplementary Figure. 5. Increased PI3K/Akt signaling does not restore developmental defects caused by GFAT1 loss.

a Thymocytes were harvested from 5-wk-old male and female WT, GFAT1$^{T-/-}$ and GFAT1/PTEN$^{T-/-}$ mice, surface-stained for CD4, CD8α, CD3ε, NK1.1, B220, Ter119, Gr1, CD25, CD44, γδTCR and TCRβ, followed by intracellular staining for phospho-T308-Akt (pT308-Akt) mAb or control IgG and analyzed by flow cytometer. Representative FACS plots show relative amount of pT308-Akt^{+} subsets. Shown is a representative experiment of three with similar results. Bar graphs represent median fluorescence intensity (MFI) of pT308-Akt phosphorylation of the indicated subset (n=6-9 mice each). Data are mean $\pm$ SD. $*p < 0.05$, $****p < 0.0001$ using one-way ANOVA followed by Tukey's *post-hoc* test. **b** Thymocytes from male and female WT and GFAT1/PTEN$^{T-/-}$ mice were lysed and protein extracts were resolved by SDS-PAGE and subjected to immunoblotting using the indicated antibodies. Closest MW (KD) markers are indicated. Representative results of 3 independent experiments with similar results are shown. **c** Thymocytes from male and female WT and GFAT1$^{T-/-}$ or GFAT1/PTEN$^{T-/-}$ mice were stained for CD4, CD8α, CD3ε, γδTCR and TCRβ, then analyzed by flow cytometer. The relative amounts of subsets are indicated. Representative FACS plots of six experiments with similar results are shown. Source data are available for a-c.

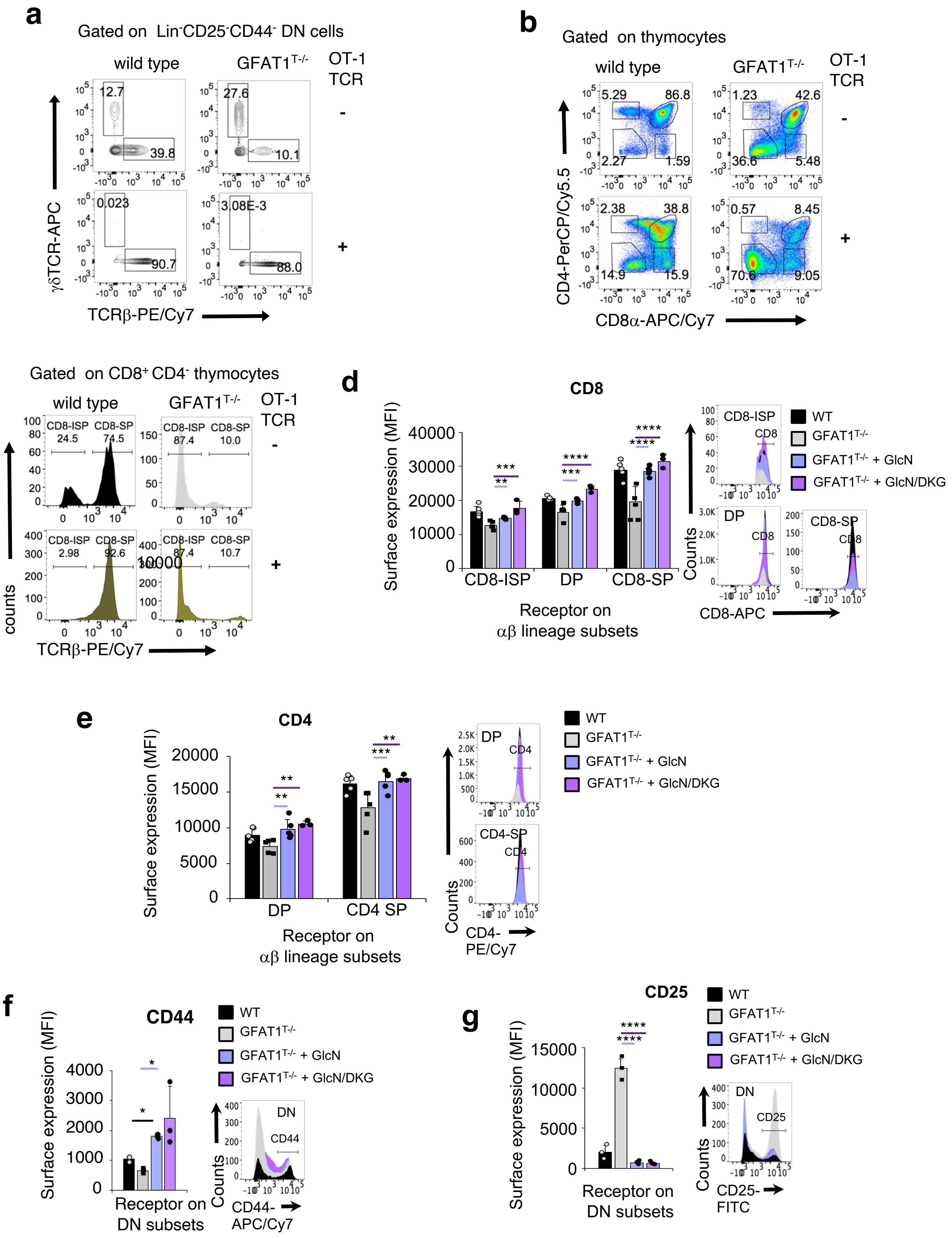
a
Gated on Lin-CD25-CD44- DN cells
wild type
GFAT1T-/-
OT-1 TCR
-
+
12.7
39.8
27.6
10.1
0.023
90.7
3.08E-3
88.0
γδTCR-APC
TCRβ-PE/Cy7
b
Gated on thymocytes
wild type
GFAT1T-/-
OT-1 TCR
-
+
5.29
86.8
2.27
1.59
1.23
42.6
36.6
5.48
2.38
38.8
14.9
15.9
0.57
8.45
70.6
9.05
CD4-PerCP/Cy5.5
CD8α-APC/Cy7
c
Gated on CD8+ CD4- thymocytes
wild type
GFAT1T-/-
OT-1 TCR
-
+
CD8-ISP
CD8-SP
24.5
74.5
87.4
10.0
2.98
92.6
87.4
10.7
counts
TCRβ-PE/Cy7
d
CD8
Surface expression (MFI)
CD8-ISP
DP
CD8-SP
Receptor on αβ lineage subsets
CD8-ISP
DP
CD8-SP
CD8
Counts
CD8-APC
WT
GFAT1T-/-
GFAT1T-/- + GlcN
GFAT1T-/- + GlcN/DKG
e
CD4
Surface expression (MFI)
DP
CD4 SP
Receptor on αβ lineage subsets
DP
CD4-SP
CD4
Counts
CD4-PE/Cy7
WT
GFAT1T-/-
GFAT1T-/- + GlcN
GFAT1T-/- + GlcN/DKG
f
CD44
Surface expression (MFI)
Receptor on DN subsets
DN
CD44
Counts
CD44-APC/Cy7
WT
GFAT1T-/-
GFAT1T-/- + GlcN
GFAT1T-/- + GlcN/DKG
g
CD25
Surface expression (MFI)
Receptor on DN subsets
DN
CD25
Counts
CD25-FITC
WT
GFAT1T-/-
GFAT1T-/- + GlcN
GFAT1T-/- + GlcN/DKG

Supplementary Figure 6 Dietary supplementation with Glucosamine and DKG, but not overexpression of OT-1 TCR, partly restores αβ-T cell development.
a Thymocytes were harvested from 5-wk-old male and female WT, GFAT1$^{T-/-}$, transgenic OT-1TCR and OT-1TCR/GFAT1$^{T-/-}$ mice, stained for Lin, CD4, CD8α, CD25, CD44, Vα2, TCRβ, γδTCR and analyzed by flow cytometer. FACS plots depict proportion of Lin^{-}CD25^{-}CD44^{-} DN thymocytes expressing either γδTCR or TCRβ on their surface in the presence or absence of a transgenic Vα2/Vβ5, OT-1TC. Results shown are representative of three experiments with similar results.
b-c. Thymocytes were harvested from 5-wk-old male and female WT, GFAT1$^{T-/-}$, transgenic OT-1TCR and OT-1TCR/GFAT1$^{T-/-}$ mice, stained for CD4, CD8α, CD3ε, Vα2, TCRβ, γδTCR and analyzed by flow cytometer. FACS plots show proportion of CD4 vs CD8 subsets in wt or GFAT1-deficient thymocytes expressing or not the transgenic OT-1 TCR (b) or immature TCRβ^{low} CD8-ISP vs mature TCRβ^{high} CD8-SP thymocytes expressing or not the transgenic OT-1 TCR (c**).** Relative numbers of subsets are indicated. Shown are representative of three experiments with similar results.
d-g Thymii were surgically removed from WT and GFAT1$^{T-/-}$ e18/19 embryos and one lobe was cultured for 7 days in complete media containing glucosamine (GlcN) with or without dimethyl-2-ketoglutarate (DKG) and the adjoining lobe was cultured in complete media only as control. Thymocytes were harvested, stained for CD4, CD8, CD25, CD44, TCRβ and γδTCR and analyzed by flow cytometer. Bar graphs represent median fluorescence intensity (MFI) of CD8 levels on the surface of CD8-ISP, DP and CD8-SP cells (n= 3-5 mice each)(d); CD4 levels on the surface of DP and CD4-SP thymocytes (n=3-5mice each)(e); CD44 levels on the surface of DN thymocytes (n=3 mice each)(f); CD25 levels on the surface of DN thymocytes (n=3 mice each). Data are mean $\pm$ SD. **p*<.05, ***p* < 0.01, ****p* < 0.001, *****p*<0.0001 using one-way ANOVA followed by Tukey's (d,e,g) or Sidak's (f) *post-hoc* test. Respective representative FACS plots are from at least three experiments with similar results. Source data are available for a-g.

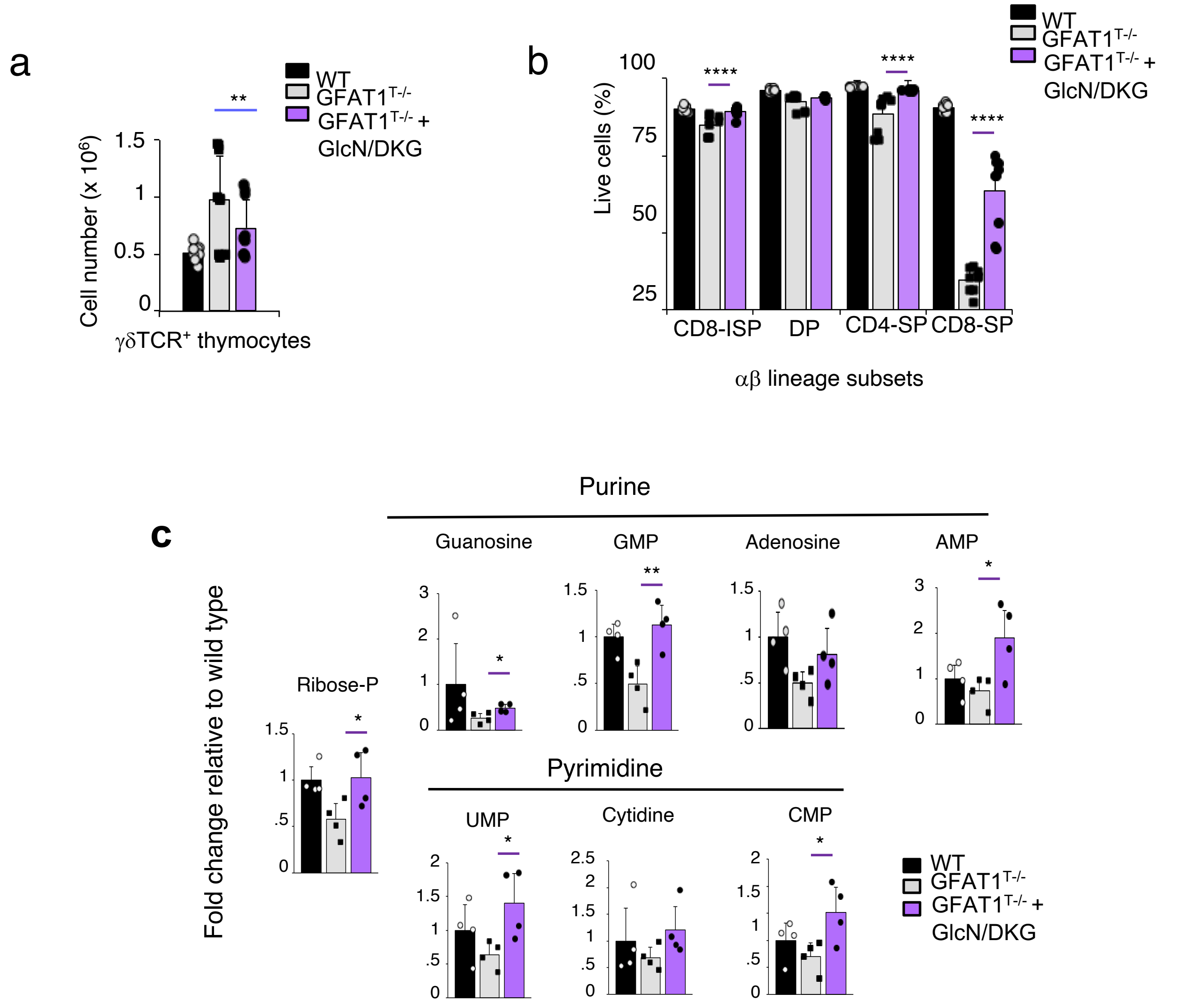

Supplementary Figure 7 Dietary supplementation of GFAT1$^{T-/-}$ mice with glucosamine and dimethyl-2-ketoglutarate partially restores αβ-thymocyte development by increasing nucleotide levels and cell viability.

3-wk-old male and female WT or GFAT1$^{T-/-}$ littermates were fed with regular water or water containing GlcN and DKG (100 mM each). After 1 month, thymocytes were harvested. **a-b** Cells were stained for CD4, CD8α, CD147, TCRβ, γδTCR, Annexin V and analyzed by flow cytometer. Bar graph shows total number of γδ-thymocytes that were obtained by multiplying the total thymocyte number by the percentage of thymocytes expressing γδTCR on their surface (n= 3 mice each with 3 technical replicates each)(a) and the proportion of live (Annexin V$^-$) CD8-ISP, DP, CD4-SP and CD8-SP thymocytes (n=.3 mice each with 3 technical replicates each)(b). Data (a, b) are mean ± SD. *p < 0.05, **p < 0.01, ***p < 0.001 using one-way ANOVA followed by Sidak's *post-hoc* test. **c** Metabolites were isolated from thymocytes analyzed by LC/MS. Bar graphs represent fold changes of indicated metabolite relative to WT (n=4 independent samples each). Data are mean ± SD. *p < 0.05, **p < 0.01, ***p < 0.001 using one-way ANOVA followed by Sidak's *post-hoc* test. Source data are available for a-c.

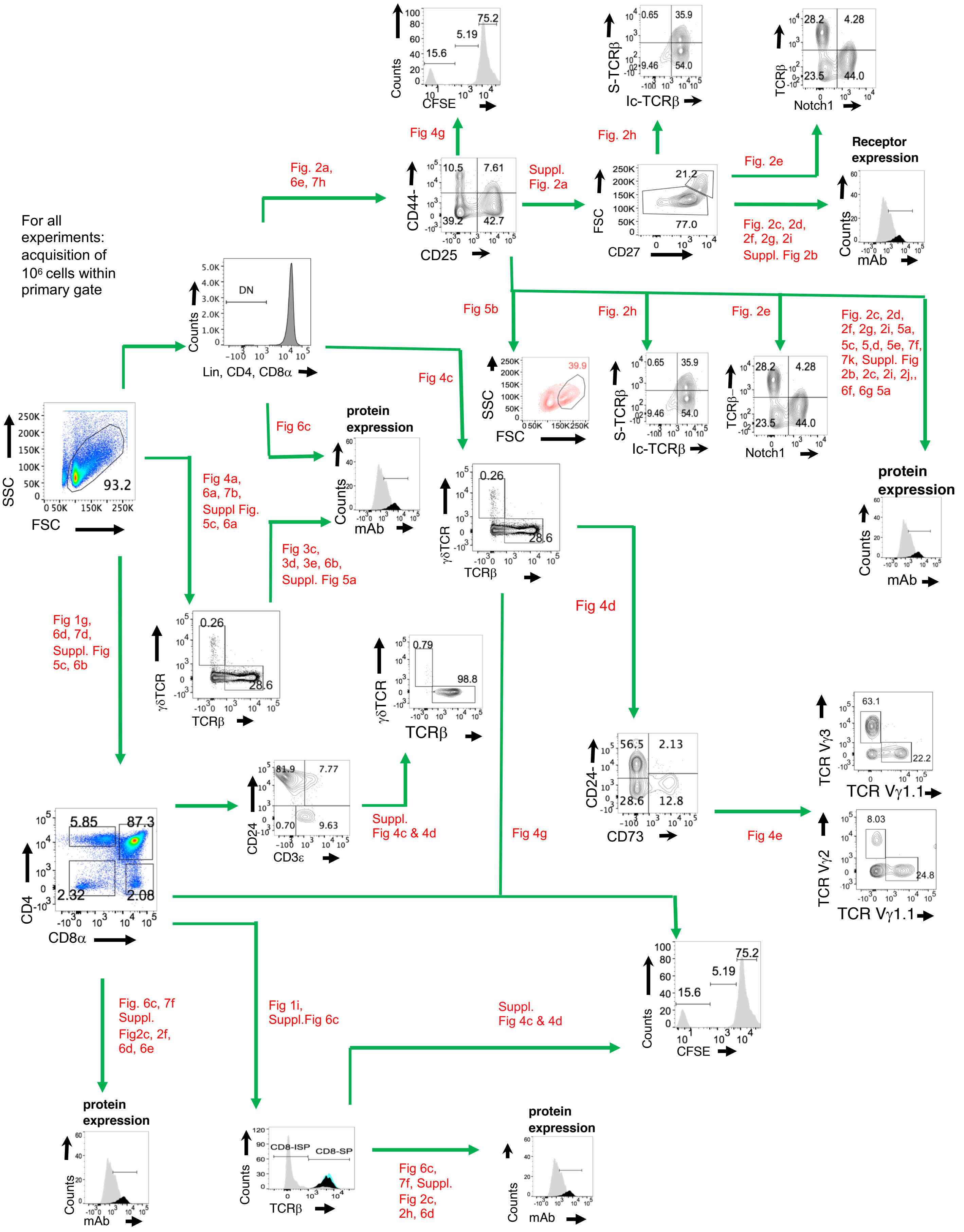

Supplementary Fig. 8. Gating Strategy, Thymocytes

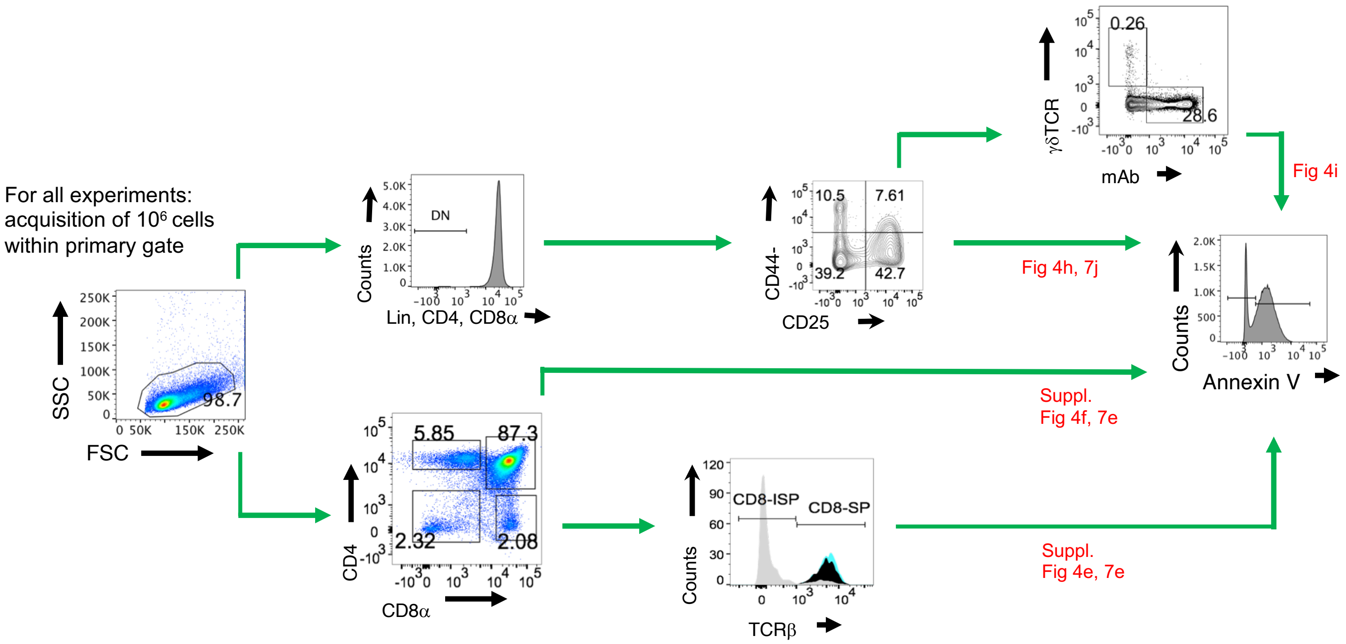

Supplementary Fig. 9. Gating Strategy, Thymocytes: Analysis of cell death

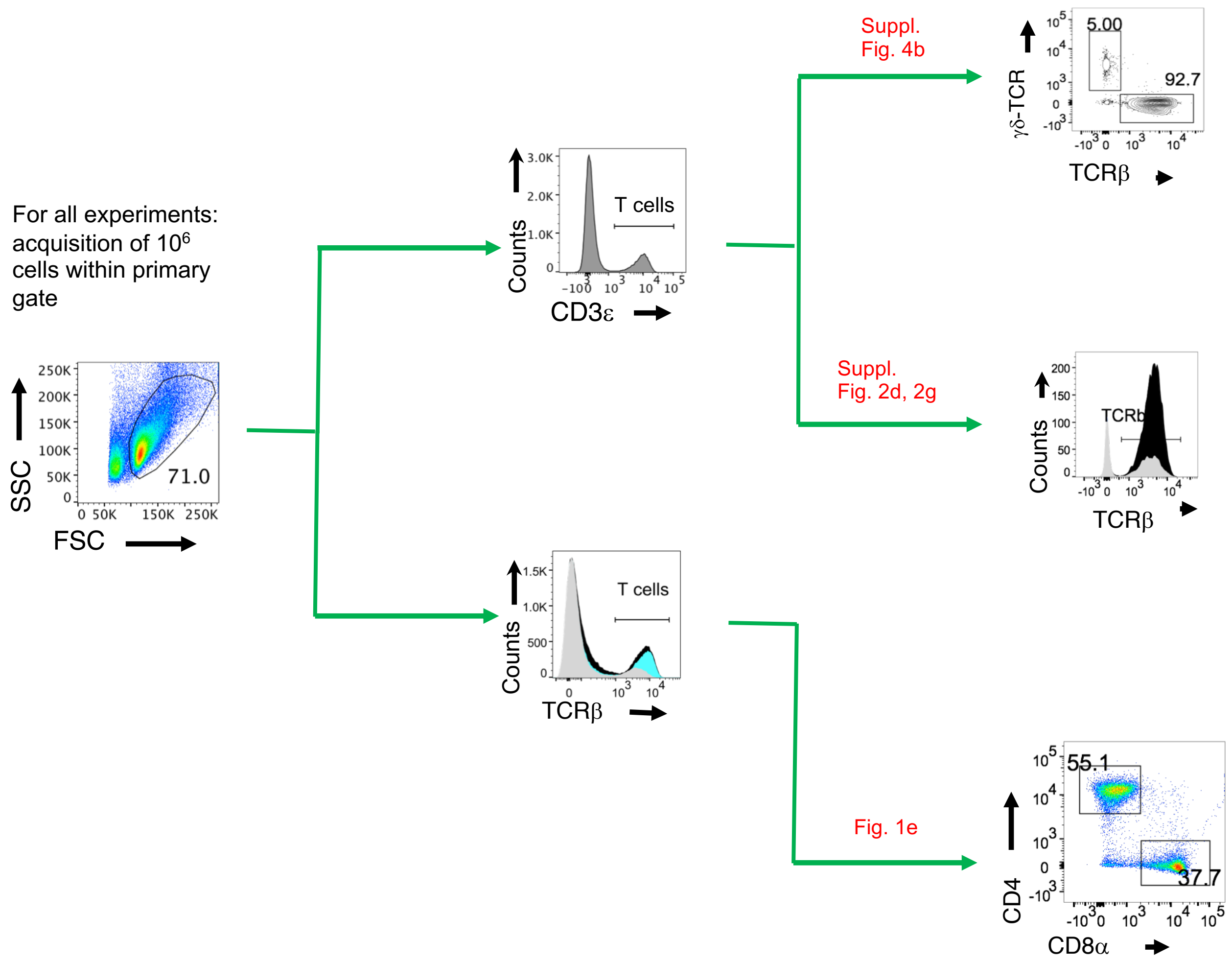

Supplementary Fig. 10. Gating Strategy, Splenocytes

Supplementary Table 1: Primer sequences used for mice genotyping and gene expression by RT-PCR.

gene	Forward primer (5'- 3')	Reverse primer (5'- 3')	Band (bp)
GFAT1$^{+/+}$	GGTGTTAACAGGGAGCCATC	GCTCCCGTTCCAATACTCAA	~290
GFAT1$^{f/f}$	GGTGTTAACAGGGAGCCATC	GCTCCCGTTCCAATACTCAA	~400
Lck-Cre	CCTTGGTGGAGGAGGGTGGAATGAA	AATGTTGCTGGATAGTTTTTACTGC	~600
Lck	CCTTGGTGGAGGAGGGTGGAATGAA	TAGAGCCCTGTTCTGGAAGTTACAA	348
Rictor$^{+/+}$	TTATTAACTGTGTGTGGGTTG	CGTCTTAGTGTTGCTGTCTAG	197
Rictor$^{f/f}$	TTATTAACTGTGTGTGGGTTG	CGTCTTAGTGTTGCTGTCTAG	295
PTEN$^{+/+}$	CAAGCACTCTGCGAACTGAG	AAGTTTTTGAAGGCAAGATGC	156
PTEN$^{f/f}$	CAAGCACTCTGCGAACTGAG	AAGTTTTTGAAGGCAAGATGC	328
	Primers below were used for RT-PCR		
TCRβ	CAGCACGGACCCTCAGGCCTA	TTTGGGTGAGCCCTCTGGCCA	
CD25	AACGGGGTGGACTCAGCCCC	CCAGGTGAGCCCGCTCAGGA	
rictor	CTAGGTGGCATTGACATTCAGC	CTAGGAAACAAGGAAGCATTCAG	
GAPDH	GATTGTTGCCATCAACGACC	CCATGGTGGTGAAGACACCA	

Supplementary Table 2: Antibodies used for immunoblotting

Antigen	clone	Catalog #	vendor
GFAT1	EPR4854	ab125069	Abcam, MA
Rictor	53A2	2114	Cell Signaling Technology, MA
mTOR	7C10	2983	Cell Signaling Technology, MA
PTEN	138G6	9559	Cell Signaling Technology, MA
Akt		9272	Cell Signaling Technology, MA
phospho-Akt (S473)	D9E	4060	Cell Signaling Technology, MA
GLS		12855-1-AP	Proteintech Group, IL
CAD	D2T8H	93925	Cell Signaling Technology, MA
LDHA		2012	Cell Signaling Technology, MA
phospho-Y10-LDHA		8176	Cell Signaling Technology, MA
HIF-1α	D2U3T	14179	Cell Signaling Technology, MA
eIF2α	D7D3	5324	Cell Signaling Technology, MA
phospho-S51-eIF2α	D9G8	3398	Cell Signaling Technology, MA
IRE1α	14C10	3294	Cell Signaling Technology, MA
ATF4	D4B8	11815	Cell Signaling Technology, MA
BIP	C50B12	3177	Cell Signaling Technology, MA
TCRβ	H197	sc9101	Santa Cruz Biotechnology, CA
β-actin	C4	sc-47778	Santa Cruz Biotechnology, CA

Supplementary Table 3: Antibodies used for flow cytometry

Antigen	Clone	Catalogue number	Vendor
TCRβ	H57-597	109208,109227,109222	Biolegend (San Diego, CA)
γδTCR	GL3	118118	Biolegend (San Diego, CA)
Vα2	B20.1	127820	Biolegend (San Diego, CA)
Vβ5	MR9-4	139504	Biolegend (San Diego, CA)
CD3ε	145-2C11	100330,100312	Biolegend (San Diego, CA)
CD4	RM4-5	100539	Biolegend (San Diego, CA)
CD5	53-7.3	100623	Biolegend (San Diego, CA)
CD8α	53.6.7	100714,100722	Biolegend (San Diego, CA)
CD44	IM7	103032,103028	Biolegend (San Diego, CA)
CD24	M1/69	101813	Biolegend (San Diego, CA)
CD73	TY/11.8	127223	Biolegend (San Diego, CA)
CD25	PC61	102015,102030	Biolegend (San Diego, CA)
CD98	4F2	128210	Biolegend (San Diego, CA)
CD127	A7R34	135039	Biolegend (San Diego, CA)
CD147	OX-114	123717	Biolegend (San Diego, CA)
NK1.1	PK136	108703	Biolegend (San Diego, CA)
B220	RA3-6B2	103212	Biolegend (San Diego, CA)
Ter119	TER-119	116212	Biolegend (San Diego, CA)
Gr1	RB6-8C5	108411	Biolegend (San Diego, CA)
CD27	LG.3A10	124226	Biolegend (San Diego, CA)
CXCR4	L276F12	153805	Biolegend (San Diego, CA)
Notch1	HMN1-12	130613	Biolegend (San Diego, CA)
Vγ1.1/CR4	2.11	141103,141104	Biolegend (San Diego, CA)
Vγ2	UC3-10A6	137703	Biolegend (San Diego, CA)
Vγ3	536	137503	Biolegend (San Diego, CA)
phospho-S473-Akt	D9E	4075	Cell Signaling Technology, MA
phospho-T308-Akt	D25E6	48646	Cell Signaling Technology, MA
phospho-T202/Y204-ERK	4B11B69	675507	Biolegend (San Diego, CA)
GluT1	EPR3915	ab195020	Abcam, MA
O-GlcNAc	RL2	ab2739	Abcam, MA
Purified CD16/32	93	101302	Biolegend (San Diego, CA)
