# Supplementary material for: Dietary glucosamine overcomes the defects in αβ-T cell ontogeny caused by the loss of de novo hexosamine biosynthesis
Source: Nat Commun. 2022 Dec 1;13:7404. doi: 10.1038/s41467-022-35014-w (PMC9715696; doi:10.1038/s41467-022-35014-w)
Supplement: Supplementary file 3 — Reporting Summary [file 41467_2022_35014_MOESM3_ESM.pdf]

nature portfolio

Corresponding author(s): Estela Jacinto and Guy Werlen

Last updated by author(s): 2022/11/01

Reporting Summary

Nature Portfolio wishes to improve the reproducibility of the work that we publish. This form provides structure for consistency and transparency in reporting. For further information on Nature Portfolio policies, see our Editorial Policies and the Editorial Policy Checklist.

Statistics

For all statistical analyses, confirm that the following items are present in the figure legend, table legend, main text, or Methods section.

n/a	Confirmed	
☐	☒	The exact sample size (*n*) for each experimental group/condition, given as a discrete number and unit of measurement
☐	☒	A statement on whether measurements were taken from distinct samples or whether the same sample was measured repeatedly
☐	☒	The statistical test(s) used AND whether they are one- or two-sided<br>*Only common tests should be described solely by name; describe more complex techniques in the Methods section.*
☒	☐	A description of all covariates tested
☒	☐	A description of any assumptions or corrections, such as tests of normality and adjustment for multiple comparisons
☐	☒	A full description of the statistical parameters including central tendency (e.g. means) or other basic estimates (e.g. regression coefficient) AND variation (e.g. standard deviation) or associated estimates of uncertainty (e.g. confidence intervals)
☐	☒	For null hypothesis testing, the test statistic (e.g. *F*, *t*, *r*) with confidence intervals, effect sizes, degrees of freedom and *P* value noted<br>*Give P values as exact values whenever suitable.*
☒	☐	For Bayesian analysis, information on the choice of priors and Markov chain Monte Carlo settings
☒	☐	For hierarchical and complex designs, identification of the appropriate level for tests and full reporting of outcomes
☒	☐	Estimates of effect sizes (e.g. Cohen's *d*, Pearson's *r*), indicating how they were calculated

Our web collection on statistics for biologists contains articles on many of the points above.

Software and code

Policy information about availability of computer code

Data collection	Mice genotyping was performed by PCR using a PTC-200 DNA Engine thermal cycler from MJ Research/BioRad<br>Flow cytometry data were collected on a BD FACS Verse flow cytometer using the BD FACSuite Software (V1.0.3)<br>Immunoblots were visualized using the Imager 600 from Amersham<br>Metabolites were collected using HILIC chromatography coupled with negative mode electrospray ionization to Q Exactive PLUS Orbitrap mass spectrometer<br>Proteomics data were collected by nanospray LC-MS/MS using a Thermo Q Exactive HS mass spectrometer<br>QRT-PCR data were generated on a QuantStudio 3 Real-Time PCR system from Applied Biosystems
Data analysis	PCR fragments were analyzed on 2% agarose gels<br>Flow cytometry data were analyzed using FlowJo (TreeStar) software (Version V10.0.8)<br>Image J software, version 1.51 (100),was used for densitometric analysis of immunoblots<br>Quantitative proteomics analysis was performed using Ingenuity Pathway Analysis (Qiagen, version 01-20-04).<br>Metabolomics was analyzed using Maven software (Build 682).<br>Histograms and statistical analysis were performed with excel software from Microsoft, version 16.16.23 (200615) and GraphPad Prism version 9.4.1<br>Glycan structures were assigned using Glycoworkbench software (version 2.1 stable [Build 157]) based on precursor masses (Sodiated) and common mammalian biosynthetic pathway.

For manuscripts utilizing custom algorithms or software that are central to the research but not yet described in published literature, software must be made available to editors and reviewers. We strongly encourage code deposition in a community repository (e.g. GitHub). See the Nature Portfolio guidelines for submitting code & software for further information.

Data

Policy information about availability of data

All manuscripts must include a data availability statement. This statement should provide the following information, where applicable:

- Accession codes, unique identifiers, or web links for publicly available datasets
- A description of any restrictions on data availability
- For clinical datasets or third party data, please ensure that the statement adheres to our policy

All data are included in the Supplementary Information or Source Data. The raw numbers for charts and graphs are available in the Source Data file whenever possible. Proteomics dataset have been deposited in MassIVE with the accession code MSV000089842

Field-specific reporting

Please select the one below that is the best fit for your research. If you are not sure, read the appropriate sections before making your selection.

☒ Life sciences ☐ Behavioural & social sciences ☐ Ecological, evolutionary & environmental sciences

For a reference copy of the document with all sections, see nature.com/documents/nr-reporting-summary-flat.pdf

Life sciences study design

All studies must disclose on these points even when the disclosure is negative.

Sample size	No statistical method was used to predetermine sample size.. Based on several years of working with most of our current animal models, we have taken into consideration that smaller number of cells will be obtained in GFAT1, GFAT1/PTEN and rictor knockout mice with or without transgenic TCR. Therefore, we needed more of these mice since we have done biochemical analyses as well as metabolomics, proteomics and therefore needed to obtain sufficient amount of cells. Typically the amount of thymocytes harvested from a 5 wk-old WT thymus is equivalent to ~ 3-4 thymus from rictor-deficiency and 4-5 thymus from GFAT1- or any combination with GFAT1-deficiency (cf. Chou et all, J Immunol 2014; 193:1162-1170 and current manuscript). Experiments are typically done using 3-6 of each cohort to get statistical significance. A p value of less than 0.05 was considered significant.
Data exclusions	No data were excluded
Replication	Each experiments were performed 2-4 times for reproducibility. All attempts at replication were successful. Glycomics experiment was only performed once on pooled samples comparing WT vs GFAT1-deficient samples.
Randomization	Samples were allocated based on mouse genotyping done by PCR
Blinding	Blinding was not relevant for the majority of our studies since quantitative analysis was performed to compare samples. For FTOCs supplementation, analysis was blinded since genotype sof samples were only revealed after results of supplementation effects were obtained.

Reporting for specific materials, systems and methods

We require information from authors about some types of materials, experimental systems and methods used in many studies. Here, indicate whether each material, system or method listed is relevant to your study. If you are not sure if a list item applies to your research, read the appropriate section before selecting a response.

Materials & experimental systems

n/a	Involved in the study	
☐	☒	Antibodies
☒	☐	Eukaryotic cell lines
☒	☐	Palaeontology and archaeology
☐	☒	Animals and other organisms
☒	☐	Human research participants
☒	☐	Clinical data
☒	☐	Dual use research of concern

Methods

n/a	Involved in the study	
☒	☐	ChIP-seq
☐	☒	Flow cytometry
☒	☐	MRI-based neuroimaging

Antibodies

Antibodies used	,Antibodies used for Flow cytometry:the following are all from Biolegend: TCRalpha (clone H57-597; Cat#109208, 109227, 109222), gamma/deltaTCR (clone GL3,Cat#118118), Valpha2 (clone B20.1, Cat#127820), Vbeta5(clone MR9-4), CD3epsilon (clone 145-2C11, Cat#100330, 100312), CD4 (clone RM4-5, Cat#100539), CD8alpha(clone 53.6.7, Cat# 100714, 100722), CD25 (clone PC61,

Cat#102015, 102030), CD27 (clone LG.3A10, Cat# 124226), CD44 (clone IM7, Cat#103032, 103028), CD98 (clone 4F2, Cat# 128210), CD127 (clone A7R34, Cat#135039), CD147 (clone OX-114, Cat#123717), NK1.1 (clone PK136), B220 (clone RA3-6B2), Ter119 (clone TER-119), Gr1 (clone RB6-8C5), CXCR4 (clone QA16A08), Notch1 (clone HMN1-12, Cat# 130613), phosphor-T202/Y204-ERK (clone 4B11B69), purified CD16/32 (clone 93) from Biolegend. Phosphor-S473-Akt (clone D9E, Cat# 4075), phosphor-T308-Akt (clone D25E6, Cat# 48646) from Cell Signaling Technology. GluT1 (clone EPR3915, Cat# ab195020), O-GlcNAc (clone RL2,Cat#ab2739) from Abcam., CD73 (clone TY/11.8, Cat#127223) from Biolegend, CD24 (clone M1/69) from Biolegend, Vgamma1.1/CR4 (clone 2.11, Cat# 141103, 141104) from Biolegend, Vgamma2 (clone UC3-10A6, Cat# 137703) from Biolegend, Vgamma3 (clone 536, Cat#137503) from Biolegend

Antibodies used for immunoblotting: GFAT1 (clone EPR4854, Cat#ab125069) from Abcam. The following are all from Cell Signaling Technology: Rictor (clone 53A2, Cat#2114), mTOR (clone 7C10, Cat#2983), PTEN (clone 138G6, Cat#9559), CAD (clone D2T8H, Cat#93925), phosphor-S473-Akt (clone D9E, Cat#4060), Hif-1alpha (clone D2U3T, Cat#14179), eIF2alpha (clone D7D3, Cat#5324), phosphor-S51- eIF2alpha (clone D9G8, Cat#3398), IRE1alpha(clone 14C10, Cat#3294), ATF4 (clone D4B8, Cat#11815), BIP (clone C50B12, Cat#3177), Akt (cat # 9272), LDHA (cat# 2012), phosphor-Y10-LDHA (cat# 8176) . GLS (cat# 12855-1-AP) from Proteintch Group. TCRbeta (clone H197, Cat#), Beta-actin (clone AC-74) from Santa Cruz Biotechnology.

Validation	All antibodies used have been specifically validated by the manufacturers. Immunoblotting antibodies have been validated by manufacturers by performing immunoblotting of cell extracts that either express (positive control) or not express (negative control) the protein of interest. Antibodies for immunostaining and flow cytometry have been validated by the manufacturers by performing immunostaining of relevant cells using the specific antibody or control isotype followed by flow cytometry.

Animals and other organisms

Policy information about studies involving animals; ARRIVE guidelines recommended for reporting animal research

Laboratory animals	Homozygous C57BL/6 Gfat1f/f (Mary Lyon Center Harwell Science and Innovation Center, UK), C57BL/6 rictorf/f (Bentzinger et al. 2008, Cell Metab.) or C57BL/6 Ptenf/f (Jackson Labs; Strain C;129S4-Ptentm1Hwu/J) mice were crossed with heterozygous C57BL/6 Lck-Cre animals (Taconic farms, NY), which generates WT as well as T-cell-specific Gfat1 (GFAT1T-/-), rictor (rictorT-/-) or Pten (PTENT-/-)knockout mice owing to the expression of Cre under the control of the proximal promoter of Lck. GFAT1/PTENT-/- double knockout mice were obtained by crossbreeding GFAT1T-/- and PTENT-/- animals for several generations. To specifically delete GFAT1 in the OT-1 TCR background, we crossed C57BL/6/OT-1 mice (Werlen et al. 2000, Nature) with homozygous GFAT1T-/- to obtain OT-1/GFAT1T-/- animals. All mice were genotyped by PCR using the respective primers: GGTGTTAACAGGGAGCCATC, GCTCCCGTTCCAATACTCAA for GFAT1. TTATTAACTGTGTGTGGGTTG, CGTCTTAGTGTTGCTGTCTAG for rictor. CAAGCACTCTGCGAACTGAG, AAGTTTTTGAAGGCAAGATGC for PTEN. CCTTGGTGGAGGAGGGTGGAATGAA, AATGTTGCTGGATAGTTTTTACTGC for Lck-CRE. CCTTGGTGGAGGAGGGTGGAATGAA, TAGAGCCCTGTTCTGGAAGTTACAA for Lck.
Wild animals	No wild animals were used in the study
Field-collected samples	No field-collected samples were used in the study.
Ethics oversight	Handling and experimentation protocols have been reviewed and used in accordance with IACUC regulations of Rutgers University

Note that full information on the approval of the study protocol must also be provided in the manuscript.

Flow Cytometry

Plots

Confirm that:

- [x] The axis labels state the marker and fluorochrome used (e.g. CD4-FITC).
- [x] The axis scales are clearly visible. Include numbers along axes only for bottom left plot of group (a 'group' is an analysis of identical markers).
- [x] All plots are contour plots with outliers or pseudocolor plots.
- [x] A numerical value for number of cells or percentage (with statistics) is provided.

Methodology

Sample preparation	Thymocytes and peripheral T cells harvested in complete DMEM from mice thymus and spleen, respectively were counted by trypan blue exclusion. Cells were incubated with anti-CD16/32 to block non-specific antibody binding followed by staining for receptor expression using antibodies as listed above. Stained cells were analyzed by flow cytometry, or further fixed and permeabilized with BD Cytofix/Cytoperm reagents according to the manufacturer's protocol. Permeabilized cells were then stained for intracellular protein expression, phosphorylation or O-GlcNAcylation and analyzed by flow cytometry. To analyze cell proliferation, thymocytes were labeled with 2µM of CFSE (Sigma-Aldrich) prior to culturing in complete DMEM media at 37°C for up to 48 hrs. Cells were collected at 0, 24 or 48h, stained and cell division was analyzed on the flow cytometer by monitoring the shift in CFSE fluorescence. To assess cell viability, ex vivo-cultured thymocytes were stained with Annexin V (BD Pharmingen, CA) and propidium iodide (PI) (Sigma-Aldrich) prior to flow cytometric analysis.
Instrument	BD FACS Verse with BD FACSuite software (V1.0.3)
Software	BD FACSuite software for acquisition. FlowJo software (TreeStar) for analysis (V10.0.8)

Cell population abundance	To avoid acquisition of no specific cells, all cells were incubated with anti-CD16/32 to prior to staining with antibodies of interest. 100000 events were acquired in the initial FSC/SSC gate (85-95% of all events). Proportions of further gated cells are indicated in the plots of each respective figures.
Gating strategy	Only live cells from the FSC/SSC gating were considered, excluding dying cells with very low FSC/SSC or dead cells with very high SSC staining. Live splenocytes were gated for CD3epsilon expressing T cells. T cells were further stained for TCRbeta, CD4, CD8alpha and gama/deltaTCR to further analyze the distinct peripheral T cell populations.<br>Live thymocytes were gated for TCRbeta vs gama/deltaTCR or CD4 vs CD8alpha expressing cells.<br>CD147 staining discriminates CD8-ISP thymocytes (CD8alpha+ CD147+ TCRbeta low) among CD8+ cells from CD8-SP (CD8alpha+ CD147- TCRbeta high).<br>For CD4-CD8- Lin- DN thymocyte gating, we excluded cells expressing the lineage markers NK1.1 (NK cell marker), B220 (B cell marker), Ter119 (erythroid marker), Gr1 (neutrophil marker), CD3epsilon (T cell Marker). DN cells were further subdivided from DN1 to DN4 according to the expression of CD25 and CD44. DN3 thymocytes were subdivided into DN3a and DN3b stages according to the expression of CD27 (cf supplementary Figure 2A).<br>For intracellular staining of TCRbeta, cells were surface stained with TCRbeta-PECy7 prior to fixation and permeabilization. Cells were then incubated with TCRbeta-PE for intracellular staining of the receptor.

☒ Tick this box to confirm that a figure exemplifying the gating strategy is provided in the Supplementary Information.
